# Benefits of Targeting Proprioceptors to Improve the Dynamic Trunk Balance and Quality of Life of Patients With Cerebellar Ataxia: A Case Report

**DOI:** 10.7759/cureus.78890

**Published:** 2025-02-12

**Authors:** Anam R Sasun, Raghumahanti Raghuveer, Moh'd Irshad Qureshi

**Affiliations:** 1 Department of Neurophysiotherapy, Ravi Nair Physiotherapy College, Datta Meghe Institute of Higher Education and Research (DMIHER), Wardha, IND

**Keywords:** cerebellar ataxia, dynamic balance, mass lesion, neurophysiotherapy rehabilitation, pnf

## Abstract

Cerebellar ataxia is characterized by a variety of motor and non-motor manifestations. This case report aims to present a case of cerebellar ataxia secondary to cerebellar infarct and the pivotal role of physiotherapy in gaining patients' functional recovery, thereby improving their dynamic trunk balance and quality of life. We glance at the case of a 50-year-old man, complaining of bilateral upper and lower limb weakness with clumsy balance while sitting and walking. This case report presents neurophysiotherapy rehabilitation, specifically targeting the proprioceptors to manage the condition. Interventions to improve proprioception include proprioceptive neuromuscular facilitation (PNF), sit-ups, four-point kneeling exercises, standing with upper limb movement, standing with upper limb resisted movement, and scapular retraction and protraction. Outcome measures like the Scale of Assessment and Rating of Ataxia (SARA), Berg Balance Scale (BBS), Barthel Index (BI), Trunk Impairment Scale (TIS), and World Health Organization Quality of Life (WHO-QOL) Scale were assessed. At the end of six weeks, there was a significant improvement in clinical outcome scores. The duration of rehabilitation was five days/week for a total of six weeks. We infer that the patient's symptoms ultimately improved by physiotherapy rehabilitation. Additionally, it boosted his quality of life and increased his functional independence.

## Introduction

Cerebellar ataxia is an assortment of neurological conditions caused by damage to the cerebellum or its connections. The cerebellum is in charge of centered motor action. The cerebellum's vestibular component is essential for coordinated movements. Ataxia, dysmetria, nystagmus, dysdiadochokinesia, tremor, and cognitive inability are symptoms of cerebellar dysfunction [1]. Ataxia is divided into acquired ataxias, degenerative ataxias, and hereditary ataxias [2]. Improving the balance of the population is critical to increase community participation and lower the healthcare costs associated with unintentional falls [3].

Pharmaceutical and surgical management techniques to improve the symptoms of cerebellar ataxia are limited, and rehabilitation strategies play a pivotal role [4,5]. Exercise can reduce patients' reliance on others to perform activities [6]. Proprioceptive neuromuscular facilitation (PNF) stimulates proprioception, promotes a nerve root response, and improves functional movement using a characteristic helical or diagonal pattern. PNF techniques of emphasis include rhythmic initiation, dynamic reversal, stabilizing reversal, and rhythmic stabilization. Contract-relax and hold-relax by using muscle group facilitation, inhibition, strengthening, and relaxation seek to enhance functional movement. Muscle contractions like concentric, eccentric, and statics are applied in diagonal directions, together with progressive resistance and suitable facilitation techniques, all tailored to the specific demands of the use [7].

Even though the pelvis and trunk are identified as crucial variables in activity and are frequently impaired in patients with cerebral dysfunction, they are largely ignored or not addressed routinely in rehabilitation. PNF is a well-established treatment technique that employs functional diagonal patterns to help promote, strengthen, and control motor control in neurological rehabilitation. No physiotherapy studies have been carried out on the effect of pelvic and trunk stabilization exercises on cerebellar ataxia. The article aimed to present a case of cerebellar ataxia secondary to cerebellar tumor excision and the pivotal role of physiotherapy in improving the patient's quality of life. This case report can guide future therapists on how to treat the ataxic population.

## Case presentation

Patient information

**Figure 1:**
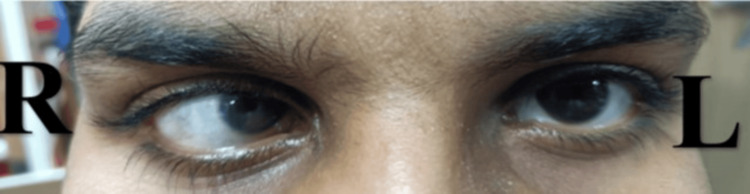
Esotropia strabismus with decreased visual acuity on the right side R: right; L: left

A 50-year-old Indian man, alcoholic for 20 years with a past surgical history of tumor excision of the right cerebellar hemisphere, visited our neurophysiotherapy outpatient department complaining of bilateral upper and lower limb weakness, slurring of speech, and clumsy balance while walking for the past three months; further, he started experiencing clumsy balance while sitting and walking. Medical history revealed the patient is an operated case of tumor excision of the right cerebellar hemisphere. This case report presents neurophysiotherapy rehabilitation, specifically targeting the proprioceptors for the management of the condition. The other associated complaints include progressive tremors in both hands, which gradually decreased his speed of writing. The tremors increased with voluntary movements of the hands and decreased with rest. The walking imbalance was first noted on uneven ground surfaces, followed by swaying on smooth surfaces. The patient also complained of diminished visual field in the right side of the eye and blurring of vision. Figure 1 depicts the patient's esotropia strabismus.

Table 1 describes the neurological examination of the patient.

**Table 1:** Neurological examination of the patient B/L: bilateral

Sr. no.	Examination	Response
1	Higher cortical functions	The patient was conscious, oriented, and attentive with an intact memory
2	Cranial nerve examination	
	Optic nerve	Esotropia strabismus with decreased visual acuity on the right side
	Abducens nerve	The activity of the lateral rectus of the right eye: absent
	Vestibulocochlear nerve	Vestibular part affected
3	Sensations	
	Superficial sensations	Light touch, pinprick, and temperature all intact
	Deep sensations	Proprioception impaired (distal toes)
	Combined cortical sensation	Intact
4	Involuntary movements	
	Tremors	Tremors in B/L hands, which decreased during rest
5	Reflexes	All superficial and deep reflexes are normal
6	Motor examination	
	Nutrition	All four limbs normal (no atrophy/hypertrophy seen)
	Muscle tone	Normal (+2 according to the Tone Grading Scale) for the B/L upper and lower limbs
	Muscle power (B/L)	Upper limb: 3/5
		Lower limb: 3/5
	Tightness	Bilateral tendon Achilles and hamstring tightness
7	Cerebellar signs	
	Dyssynergia	Present
	Dysdiadochokinesia	Present
	Dysmetria	Present
	Tremors	Present
	Nystagmus	Head titubation present
	Titubation	Present
	Speech disturbance	Slurring of speech
	Gait	Wide-based gait and decreased arm swing bilaterally
8	Balance and coordination	
	Equilibrium	Impaired (fair)
	Non-equilibrium	Impaired (Grade II)
9	Chest expansion	Axillary level: 2 cm (reduced)
		Nipple level: 1 cm (reduced)
		Xiphisternum level: 1 cm (reduced)

Therapeutic interventions

The planned rehabilitation was explored according to the neurological assessment done. Repetitive motor learning is an excellent way to gain functional recovery in cerebellar ataxia. The physiotherapy rehabilitation for weeks 1-6 is described in Table 2 and Table 3. 

**Table 2:** Physiotherapy rehabilitation for weeks 1-3 PNF: proprioceptive neuromuscular facilitation; B/L: bilateral

Goals	Interventions	Dosage
To educate patient	Education regarding the health condition physiotherapy protocol to be implemented, specifically skills to deal with the current quality of life; included stress management techniques and self-care practices	Everyday
To improve dynamic trunk balance	Trunk and pelvic PNF with rhythmic initiation will be given as well as multi-directional reach-out activities	10 repetitions×1 set
To decrease tremors in B/L hands	Limb eccentric training and dual task training	10 minutes/session
To improve postural control	Sit-ups, four-point kneeling exercises, and bridging with pelvic tilt were commenced	10 repetitions×3 sets
To decrease the tightness of the Achilles and hamstrings	Stretching for bilateral tendon Achilles and hamstrings	5 repetitions×30-second hold with 10-second rest
To improve balance	Exercises like sitting with eyes closed, sitting with neck and trunk rotation, static standing with feet apart, static balance with feet together, and eyes closed-open were done	10 repetitions×3 sets
To improve gaze stability	Gaze stabilization, habituation exercises, imaginary target exercises, walking with head movements, standing gaze stabilization, vestibuloocular reflex exercises	10 repetitions×2 sets

**Table 3:** Physiotherapy rehabilitation for weeks 4-6 PNF: proprioceptive neuromuscular facilitation

Goals	Interventions	Dosage
To maintain postural control	Here, further progression in exercises was done, which included standing with upper limb movement, standing with upper limb resisted movement, and scapular retraction and protraction	10 repetitions×2 sets
To maintain dynamic trunk balance	Further progression in trunk and pelvic PNF patterns was done, and bilateral lower extremity flexion with knee flexion for lower trunk extension, trunk lateral flexion, and right lateral flexion with extension were given. Pelvic PNF patterns were also commenced	10 repetitions×2 sets
To maintain balance	Further progression into wobble board exercises was done. Static standing balance step-tandem stance with further progression into foam was done. Single-leg standing with reaching to the floor and returning subsequently, tandem walking, sideway and backward walking with further progression to sit-to-stand, and balancing on toes were commenced	10 repetitions×2 sets
To improve coordination	Progression was made by altering the speed and range of movement	10 repetitions×1 set
For gait training	Using a treadmill was done. A 20-minute session with a progressive increase in velocity and step length was completed, and further progression to obstacle treadmill walking was done	10 repetitions×2 sets

Diagnostic assessment

**Figure 2:**
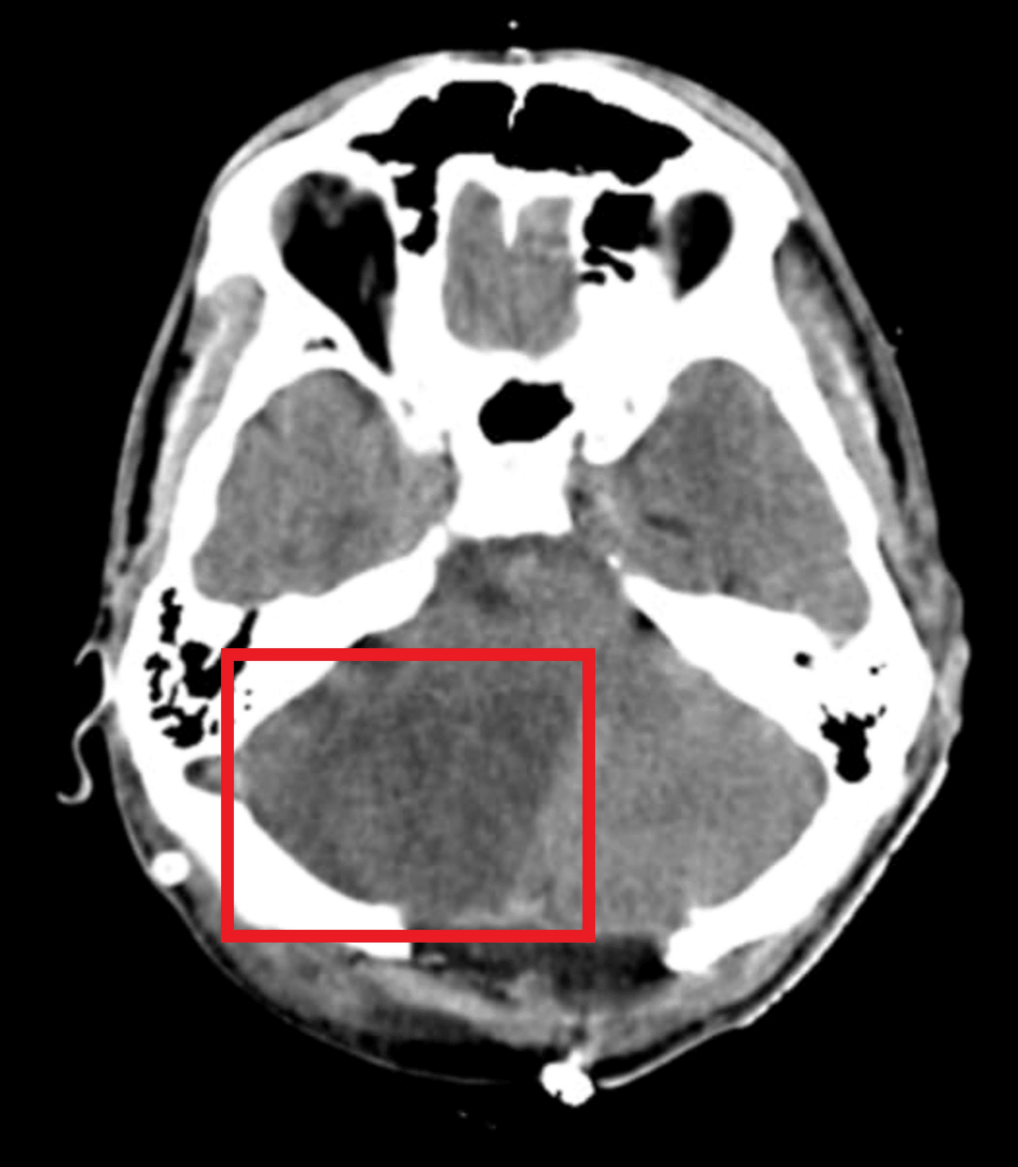
Magnetic resonance image suggestive of cerebellar infarct

There is the presence of a hypodense area in the right cerebellum, suggestive of massive right cerebellar hemisphere infarct along with cerebral edema (Figure 2).

Follow-up and outcome measures

The outcome measures were assessed on day 1 of physiotherapy rehabilitation and at the end of the sixth week. Table 4 describes the outcome measures of rehabilitation.

**Table 4:** Outcome measures of rehabilitation SARA: Scale of Assessment and Rating of Ataxia; BBS: Berg Balance Scale; TIS: Trunk Impairment Scale; BI: Barthel Index; WHO-QOL: World Health Organization Quality of Life

Sr. no.	Outcome measures	Score
1	SARA	20/40
2	BBS	30/56
3	TIS	15/23
4	BI	46/100
5	WHO-QOL Scale	80/100

A Scale of Assessment and Rating of Ataxia (SARA) score of 20/40 on day 1 indicated a moderate to high level of ataxia severity. By the end of the sixth week, improvement in SARA suggested a reduction in the severity of ataxic symptoms, demonstrating better motor control and stability. A lower SARA score post-rehabilitation would indicate enhanced coordination and reduced ataxia, translating to improved safety and independence in movements.

A Berg Balance Scale (BBS) score of 30/56 reflects a moderate risk of falls and impaired balance control. Improvement by the end of the program would suggest enhanced balance abilities, which is clinically significant as it reduces fall risk and supports functional stability. BBS scores can directly impact functional independence, as better balance supports confidence in performing activities of daily living without assistance.

A Trunk Impairment Scale (TIS) score of 15/23 indicates moderate impairment in trunk control, affecting postural stability and core strength. An increase in TIS by the sixth week would signify improved trunk stability and core engagement, essential for balance and coordinated movements. Enhanced trunk control supports safe and efficient mobility, which is particularly beneficial for patients with ataxia.

A baseline Barthel Index (BI) score of 46/100 indicates moderate dependency in activities of daily living. An improvement over rehabilitation suggests enhanced functional independence, allowing the patient to perform daily activities with less assistance, demonstrating the program's success in promoting autonomy in personal care and mobility.

A World Health Organization Quality of Life (WHO-QOL) Scale score of 80/100 suggests a relatively high quality of life. Further improvements after rehabilitation would indicate positive gains in physical, psychological, and social well-being, reflecting better overall health and independence. Higher quality of life scores also support greater satisfaction and adherence to therapy, promoting better long-term outcomes.

## Discussion

This clinical report presents the physiotherapy evaluation and rehabilitation of a patient with cerebellar ataxia secondary to an excision of a tumor at the cerebellum. Together with the patient and the patient's primary carers (wife and relatives), a physiotherapy care and treatment plan was developed. The role of physiotherapy in cerebellar ataxia is poorly understood, and there is a dearth of literature stating the position of evidence-based rehabilitation for the treatment of cerebellar ataxia. Miyai et al. published a study with evidence that backs up traditional rehabilitation programs [8]. Ayvat et al. published a study stating the impact of exergame training on patients with ataxia. They further added the additional benefits of exergame training to improve postural control [9]. Chen et al. proposed a systematic review with meta-analysis concerning the importance of transcranial direct current stimulation (tDCS) for patients with cerebellar ataxia. tDCS is domain-specific, as the recent analysis suggested a positive effect on gait and various other parameters [10].

Milne et al. conducted a study about the positive effects of computer-based training and treadmill training [11]. Also, Milne et al. proposed a protocol of conventional therapy for treating cerebellar ataxia. They included interventions like strength training, postural control, functional mobility, balance training, sensory stimulation, mobilizing, and stretching exercises and also described the beneficial effects of aquatic therapy on patients with cerebellar ataxia [12]. Weak postural reactions, trunk instability, loss of balance, and poor coordination are characteristics of children with cerebellar ataxic cerebral palsy. The relationships between the muscles of the abdomen, spine, shoulder girdle, and pelvic girdle to stabilize the trunk and offer support for movements involving the extremities were enhanced by core stability [13,14]. Sasun et al. concluded the positive effects of kinesiotaping for dysphagia in cerebellar ataxia [15].

At the end of our rehabilitation, outcome measures demonstrated a significant improvement in the patient's overall functional performance. Additionally, we also observed a decline in the severity of ataxia on the post-test scores. According to our knowledge, there is a lack of evidence concerning the effects of tailored physical therapy interventions involving trunk and pelvic PNF with traditional physical therapy exercises for cerebellar ataxia. Conclusions from the current study indicate that the patient's overall functional status improved over the next six weeks. The future implications of our study are the addition of this exercise protocol in rehabilitating patients with ataxia. Further, this study gives a future perspective for tailoring a plan of care to treat the ataxic population.

## Conclusions

The rehabilitation of patients with cerebellar ataxia requires an extensively tailored physiotherapy rehabilitation program. Many of the problems that cause patients' discomfort and affect their quality of life include not only cerebellar symptoms but also other non-motor symptoms like dysphagia, breathing difficulties, pain, and spasticity. The outcome measures used in the study showed a significant improvement in all parameters. At the end of six weeks of rehabilitation, the patient demonstrated significant improvement in motor and cerebellar symptoms, static and dynamic balance, muscle strength, and limb coordination. Physiotherapy rehabilitation is paramount in restoring physical function, alleviating discomfort, and fostering independence. It enhances both physical and emotional well-being, empowering individuals to regain control over their lives. Tailored interventions address specific impairments, promoting long-term health and preventing future complications. Ultimately, physiotherapy is indispensable in optimizing recovery and quality of life. 
